# A New and Easy Method of Applying a Tube for the Cure of the Fistula Lachrymalis

**Published:** 1781-10

**Authors:** 


V. *A new and eafy method of applying a tube for the cure of the fiftula lachrymalis. By* Jonathan Wathen, *furgeon*, F. A. S. 4to. Cadell, *London*, 1781. 16 pages, with a copper-plate, 1s.

IN cafes of fiftula lachrymalis, where the diforder has been of long continuance, and the obftruction is confiderable, furgeons are now pretty generally of opinion that an opening muft be made into the fac; in order to admit a tent of lead, or fome other folid fubftance, into the duct. The tent, whatever it be, muft be continued through the duct, and left there for the fpace of two months or more; to prevent, if poffible, a return of the obftruction. This method, however, is found by experience to be attended with great uncertainty, as the difdiſorder is frequently apt to return, in conſequence of a freſh ſwelling of the membrane which lines the duct, or of a ſponginess of the bones themſelves by which the duct is formed. The author of the work before us thinks he has remedied this imperfection in the preſent mode of treatment, by introducing a hollow tube made of gold, ſilver, or lead, into the lachrymal duct. This idea was ſuggeſted to him by obſerving that metals, not liable to ruſt, may ſafely lodge in any part of the body, for years, and even for life, without the leaſt detriment. He is aware that Heiſter long ago recommended the introduction of a metal tube in caſes of this ſort; but Heiſter conveyed his tube through a perforation of the os unguis, and of courſe his method was very different from that which is here recommended.

Mr. Wathen conſiders gold as the moſt eligible metal for the tube, becauſe it is the moſt eaſy to be preſerved in a ſtate of purity, which is the moſt important quality to be obſerved, whatever the metal be: and he prefers this or ſilver to lead, chiefly on account of their firmer texture, which muſt render a tube, made of either of them, leſs liable to be affected by any preſſure it may receive.

The

The tube is conſtructed in the following manner: Its ſhape is ſomewhat conical to prevent its deſcent into the noſe; and it is made ſufficiently long, to reach from the upper portion of the bony duct, to its aperture below the os ſpongioſum.

To introduce it more eaſily, it is furniſhed with a ſtyle, nearly as long as a common probe; the lower end of which is rounded, ſo as that, paſſing through the tube, it may exactly fill the aperture at the ſmall end: and it is prevented from paſſing further, by a ſhoulder of the ſame ſize with the outſide of the tube.

The tube is held upon the ſtyle by a doubled thread, which paſſes through a ſmall hole on one ſide of its upper or larger aperture, and is continued to a ring at the upper end of the ſtyle. By faſtening this thread to the ring, the ſtyle and tube become one inſtrument, capable of being introduced and extracted at pleaſure; and poſſeſſing all the power of a probe.

By means of this the operator, we are told, may be enabled to examine the ſtate of the diſeaſe, and to judge with the greateſt preciſion of the diameter of the duct, and conſequently of the proper ſize of the tube. For, as the duct in perſons of different ages, &c. will vary, both in diadiameter and length, there muſt be a proportional difference in the tube; for this reaſon our author recommends it to the operator to provide himſelf with ſtyles and tubes of different ſizes. He obſerves, that if the tube is too large, it cannot be introduced; and if too ſmall it will be liable to ſlip through the lower opening of the duct into the noſe; again, that if it riſes too high, it may preſs againſt the ſides of the ſac, and thus cloſe the orifice, through which the tears ſhould paſs; or that if, on the other hand, it comes down too low, it will project beyond the inferior extremity of the duct, and may produce a very troubleſome titillation. When the tube is found to fit exactly, the thread, which was paſſed through the ring at the upper end of the ſtyle, being tied in a knot, at about an inch from the top of the tube, the longer portion of it, above the knot, is directed to be cut off. By this the ſtyle will be diſengaged, ſo that it may be extracted with eaſe, leaving the tube behind, with the thread hanging out of the wound.

When the tube is fixed Mr. Wathen uſually paſſes, by ſyringe, ſome ſimple liquor through it into the noſe, as a proof of its being ſo placed that it will anſwer the intended purpoſe. The openopening made in the fac might be intirely clofed in a few days, but he thinks it right to leave the thread in for about a week, and then, if the tears, abforbed by the puncta, are conveyed by the tube to the nofe, he extracts it by cutting one fide of it with the fciffars, and drawing the other out. The little orifice through which the threads pafs will be clofed, we are told, in a few days, fo that a diforder, which had continued for months, and perhaps years, may be perfectly cured within the fhort fpace of a week.

Mr. Wathen firft performed this operation on two patients, the eldeft of whom was not more than eleven years old. In both of them the diforder was of confiderable ftanding; and in the elder had rifen to fuch a heighth, as to occafion very diftreffing apprehenfions. In one of thefe cafes the tube paffed but with little difficulty; in the other, the duct being more obftructed, the introduction was more troublefome and painful. In the latter, however, the uneafinefs foon went off after the operation was finifhed. From the time of fixing the tube, the tears, inftead of falling down the cheek, as they had been ufed to do, paffed through the artificial channel into the nofe. At the end of a week the threads were removed, and the orifice, thro'

 which which they paſſed, was healed in a day. A cure was conſequently effected in both caſes.

Since that time both our author and his partner, Mr. Ware, have repeated the operation on ſeveral perſons of different ages, and have uniformly ſucceeded in all.

After this account of his ſucceſs in this way, Mr. Wathen proceeds to obviate the objections that may be made to this proceſs. One is, that the tube may move upwards and irritate the ſac; the other, that it may paſs downwards into the noſe, and be either diſcharged or inſenſibly ſwallowed. In both theſe inſtances the operation may be ſuppoſed to fail. In reply, the author obſerves, that the tube, if of a proper ſize and well fixed, can neither aſcend nor deſcend, but that even if it ſhould chance to riſe, it may eaſily be put down again by the finger. This, he ſays, will always be found a preſent remedy, and may be repeated as often as there ſhall be occaſion, till the tube has been long enough in the duct to ſecure the continuance of the natural paſſage; after which it may be taken out by inciſion, or puſhed downward into the noſe by the probe.

With regard to the other accident to which the patient is ſuppoſed to be liable, Mr. Wathen remarks, remarks, that he has met with only one caſe, in which the tube has been diſcharged by the noſe, whereas he has known ſeveral, in which a plummet, or leathern bougie, has paſſed that way; and yet not a ſingle inſtance has occurred to him in which either the plummet, tent, or tube has been ſwallowed. But ſhould the latter circumſtance happen, as the metals, of which theſe tubes are compoſed, are not in the leaſt hurtful, the quantity uſed in them ſmall, and they are well poliſhed, he thinks it impoſſible that any harm ſhould enſue. He further adds, that the tube, although it ſhould fail of its original deſign, as an artificial duct, appears ſtill to be much more certain in its effect, and upon every account greatly preferable to the leaden tent or bougie; as it may remain longer, with leſs inconvenience, than either of thoſe ſubſtances within the lachrymal duct.

Mr. Wathen concludes his performance with a particular account of the caſe in which the tube paſſed through the noſe. The patient was a young man, 22 years of age, who had been afflicted with the diſorder from early infancy, and had nearly loſt the ſight of one eye in conſequence of repeated inflammations, which the fiſtula had occaſioned. The common mode of treating the diforder, by the introduction of a bougie, had been tried without effect. Our author paffed the tube into the duct with great eafe, and the patient continued well for about a fortnight, but at the end of that time the tube rofe in the duct. This was remedied, for a time, by preffing the tube down with the finger, but it was not long before the courfe of the tears was again obftructed, though the inftrument did not rife at all. Recollecting that it had paffed into the duct with great eafe, and that inftead of rifing, it might now be funk too low, Mr. Wathen made a fecond opening into the fac in order to remove it; and accordingly, on examination he found it was far advanced in the duct towards the nofe. The patient difcharged this tube into his handkerchief, and a fecond tube being introduced, particular attention was given that it might fit the duct more exactly than the former one had done. The tears immediately refumed their natural courfe, and continued to pafs freely through the tube for a confiderable time, but at length this fecond tube came out on blowing the nofe. This circumftance the author afcribes to the enlargement of the natural paffage, on its being perfectly healed, as the patient has remained perfectly free from his complaint ever fince.

